# Institutional time and collective behavior: end-of-week school absenteeism as a social pattern

**DOI:** 10.3389/fpsyg.2026.1852768

**Published:** 2026-07-21

**Authors:** Salem Alalwani

**Affiliations:** Department of Education and Psychology, College of Education, University of Hafr Al Batin, Hafar Al Batin, Saudi Arabia

**Keywords:** collective behavior, end-of-week absenteeism, institutional time, school absenteeism, social expectations, weekly rhythms

## Abstract

This study examines end-of-week school absenteeism as a temporally structured social phenomenon shaped by institutional time arrangements and shared social expectations. Using a mixed-methods design, the research draws on data from 411 teachers, students, and parents across several regions in Saudi Arabia. Quantitative analysis using the Relative Importance Index (RII) and qualitative thematic analysis identified eleven interrelated factors influencing absenteeism, including cumulative weekly fatigue, the organization of the school week, socially coordinated absences, digital communication practices, weak institutional supervision, and disengaging school environments. The findings show that institutional time structures and collective expectations surrounding the final day of the school week exert a strong influence on attendance behavior. These results suggest that end-of-week absenteeism should be understood not as isolated non-compliance but as a patterned social practice emerging from the interaction between institutional time regimes and everyday social life. The study contributes to interdisciplinary discussions on temporality, collective behavior, and participation within educational institutions.

## 1 Introduction

School absenteeism is a persistent challenge affecting education systems worldwide. While its causes and manifestations vary across contexts, absenteeism typically emerges from the interaction of social, cultural, institutional, and organizational factors. Persistent patterns of absenteeism disrupt instructional processes and generate negative consequences not only for students and families, but also for broader social systems. Askeland et al. (2015) identify absenteeism as one of the most pressing challenges confronting educational stakeholders due to its implications for learning outcomes, student development, and institutional effectiveness.

Beyond its educational consequences, school attendance is closely connected to broader questions of institutional sustainability and social organization. Regular participation in schooling supports the effective use of public resources and contributes to the long-term stability of educational systems. From this perspective, systematic absenteeism may weaken institutional efficiency and reduce social cohesion. Within the framework of the Sustainable Development Goals—particularly SDG 4 (Quality Education)—consistent school participation is considered essential for promoting inclusive and sustainable learning opportunities (UNESCO, 2020; Sterling, 2010). Recent research also suggests that the educational consequences of absenteeism may vary according to the timing of absences during the school year (Keppens, 2023). Previous scholarship further emphasizes that absenteeism should be understood as a complex phenomenon shaped by interacting institutional, social, organizational, and psychological influences rather than isolated individual behavior (Tonge and Silverman, 2019).

Importantly, attendance practices are not shaped solely by individual motivation or academic performance. Schooling, like many modern institutions, is organized through institutional time regimes that structure daily routines, weekly rhythms, and expectations of participation. School calendars, timetables, and the sequencing of the school week create shared temporal frameworks that influence behavior within educational settings. The end of the school week, in particular, may acquire distinct social meanings that shape collective expectations and participation patterns.

Within the Saudi educational context, absenteeism has become increasingly concentrated on Thursdays, the final day of the school week. This pattern appears consistently across elementary, middle, and secondary schools, evolving into a socially recognized phenomenon rather than isolated individual behavior. Thursday absenteeism reflects a patterned withdrawal from schooling that coincides with the temporal boundary marking the end of the institutional week. Its persistence suggests deeper interactions between institutional schedules, social expectations, and collective behavior.

While international literature often attributes absenteeism to economic hardship, political instability, or cultural marginalization, the Saudi case presents a different temporal–social configuration. Thursday absenteeism appears to be driven largely by socially coordinated practices, peer influence, and family expectations rather than structural deprivation. This distinctive pattern highlights the need to examine absenteeism not only as an educational issue but also as a temporally organized social practice embedded within weekly rhythms of social life.

Accordingly, this study examines absenteeism at the end of the school week through three interconnected dimensions. First, it situates school absenteeism within international research, emphasizing conceptual links between attendance behavior and institutional time arrangements. Second, it analyses the specific pattern of Thursday absenteeism in Saudi Arabia by drawing on national studies and observed school practices. Third, it investigates the underlying causes and perceived solutions from the perspectives of teachers, students, families, and educational stakeholders using a mixed-methods approach. By doing so, the study provides empirical insights into how temporal organization and shared social expectations shape participation within contemporary schooling systems.

This study addresses the following research questions:

1. What are the primary factors contributing to fifth-day absenteeism in Saudi schools as perceived by students, parents, and teachers?
2. What is the relative importance of the factors associated with fifth-day absenteeism in Saudi schools as perceived by students, parents, and teachers?
3. What are the perceptions of students, parents, and teachers regarding potential solutions to address fifth-day absenteeism in Saudi schools?

## 2 Literature review

### 2.1 School absenteeism in the international context

School absenteeism is widely recognized as a persistent challenge across education systems worldwide. While commonly examined in relation to academic achievement and student development, absenteeism also reflects broader social and institutional dynamics that shape participation in schooling. International research indicates that attendance patterns emerge from a complex interaction of social, economic, cultural, psychological, and organizational factors that vary across national and local contexts. Institutional structures and system-level policies play a significant role in shaping educational outcomes and patterns of student engagement across schooling contexts (Blazar and Schueler, 2024).

Askeland et al. (2015) identify absenteeism as one of the most pressing concerns facing contemporary education systems, attracting sustained attention from teachers, policymakers, and mental health professionals. Global data further illustrate the scale of the phenomenon. UNESCO Institute for Statistics (2018) reported that approximately 258 million children and adolescents were out of school worldwide, although this figure includes forms of non-enrollment and exclusion from formal education systems in addition to student absence from school. In the United States, nearly 16% of students—around 7.8 million learners—miss more than fifteen school days annually (U.S. Department of Education, 2019). These figures suggest that absenteeism extends beyond an individual behavioral issue and reflects broader institutional and educational challenges. Persistent absenteeism has increasingly been recognized as an important indicator of student risk and school performance, serving as a diagnostic tool for identifying underlying academic and social difficulties (Chang and Jordan, 2017).

International studies consistently demonstrate that persistent absenteeism is associated with long-term academic and social consequences. Research conducted in Chicago public schools found that missing fourteen or more instructional days significantly reduced students’ likelihood of graduating on time and negatively affected academic performance across subjects (Smerillo et al., 2018). Similarly, longitudinal studies show that persistent absenteeism from early childhood through middle school predicts reduced participation in post-secondary education and diminished employment prospects later in life (Ansari et al., 2020; Liu et al., 2021).

Beyond educational outcomes, absenteeism has enduring social implications. Children who experience frequent absences during primary schooling are more likely to encounter labor market difficulties, lower lifetime earnings, and reduced social mobility (Cattan et al., 2023). Persistent absenteeism has also been linked to higher dropout rates, poorer health outcomes, and increased social disadvantage in adulthood (Australian Centre for Education Statistics and Evaluation, 2022). These findings suggest that absenteeism operates as a cumulative process, producing effects that extend well beyond the schooling years. Evidence also suggests that the consequences of absenteeism extend beyond academic achievement. Using a nationally representative sample of kindergarten students in the United States, Gottfried (2014) found that persistent absenteeism was associated not only with lower reading and mathematics achievement but also with reduced educational engagement and weaker social participation.

A wide range of interrelated factors contribute to absenteeism internationally. Family-related conditions—such as conflict, separation, inconsistent discipline, and mental health challenges—frequently disrupt regular attendance (Rocque et al., 2017; Daraganova et al., 2014). In addition, parental involvement in children’s daily lives plays an important role in shaping their engagement with schooling and overall educational outcomes (Kantova, 2024). Moreover, the growing use of social media has been associated with changes in students’ emotional well-being and affective states, including stress, anxiety, and mood-related outcomes, as well as potential distractions that may influence their academic engagement (Chen and Xiao, 2022; Sumadevi, 2023). Behavioral disengagement and school climate also play a significant role, as absenteeism is closely associated with student disengagement and an increased risk of dropping out of school (Blount, 2012). In addition, empirical evidence indicates that negative or less supportive school climate environments are associated with higher levels of persistent absenteeism, highlighting the importance of students’ perceptions of their school environment in shaping attendance patterns (Van Eck et al., 2017). Importantly, research indicates that the timing and clustering of absences matter. Different forms of absence, including truancy, illness, family leave, or home-related circumstances, have distinct implications for learning, as school absenteeism represents a complex and multidimensional phenomenon encompassing diverse forms and underlying factors (Kearney et al., 2019), particularly when absences concentrate at specific points within the school calendar (Klein and Sosu, 2022; Keppens, 2023). Increasingly, absenteeism has been discussed in relation to the sustainability and organization of education systems. Persistent patterns of absence undermine the effective use of public resources, weaken human capital development, and challenge the long-term resilience of schooling institutions. From this perspective, regular attendance represents not only an educational objective but also a condition for social and institutional sustainability (UNESCO, 2020; OECD, 2020).

Overall, international evidence suggests that absenteeism should be understood as a socially patterned and institutionally embedded phenomenon rather than a series of isolated individual decisions. While existing research has documented its causes and consequences extensively, limited attention has been given to how institutional time structures—such as weekly schedules and calendar arrangements—shape attendance practices. This gap points to the need for analyses that situate absenteeism within broader temporal and organizational contexts, particularly in relation to how participation in schooling is structured over time.

### 2.2 Institutional time and weekly attendance patterns

Most research on school absenteeism has focused on individual, family, or school-related factors. Some theoretical perspectives, however, have emphasized the role of time and recurring institutional routines in shaping behavior. Bronfenbrenner and Morris (2006) described the chronosystem as the temporal dimension of human development, referring to the influence of routines, transitions, and recurring patterns over time. Within educational settings, these patterns include school schedules, the organization of the academic week, and daily institutional routines. Recent scholarship has similarly argued that school attendance problems should be examined through broader ecological and institutional frameworks rather than through isolated behavioral explanations alone (Tonge and Silverman, 2019). Previous research has shown that absenteeism is influenced not only by individual and family-related conditions, but also by broader school-level factors such as school climate, institutional routines, and students’ perceptions of the educational environment (Karlberg et al., 2022).

A report published by the Children’s Commissioner for England (2022) indicated that school absences in England were more likely to occur on Mondays and Fridays than during the middle of the school week. The report further noted that Friday absences represented the highest rates of both authorized and unauthorized absence, with attendance patterns becoming more irregular before holidays and at the end of academic terms.

Recent studies have increasingly examined when absences occur rather than focusing only on how often they occur. Keppens (2023) argued that the educational consequences of absenteeism may differ according to the timing of absences, suggesting that attendance patterns should be examined in relation to school routines and temporal organization.

Hollon et al. (2025) similarly found higher absence rates on Mondays and Fridays and linked these patterns to cumulative weekly fatigue, weekend-related family routines, elective absences before holidays, and changing attendance behaviors following the COVID-19 pandemic. The authors also reported increased absences before holidays and during the final weeks of the school year, suggesting that holiday extension and end-of-week routines may contribute to irregular attendance patterns.

Contemporary scholarship has also emphasized that problematic school absenteeism should be understood as a complex phenomenon shaped by interacting institutional, social, psychological, and organizational factors rather than isolated individual behavior (Harling and Strandler, 2025). Interest in weekly attendance patterns has also grown in recent years. Using national attendance data from England, Clifton-Sprigg and James (2025) identified what they described as a “Friday Effect,” where absence rates were consistently higher on Fridays than on other school days. The study suggested that this pattern was partly associated with families extending holidays before bank holidays and half-term breaks, as well as attempts to avoid travel congestion during peak holiday periods. The authors also found that the “Friday effect” was more pronounced in areas characterized by higher levels of persistent absenteeism and socioeconomic deprivation.

Kirksey et al. (2023) found that students were significantly more likely to experience unexcused absences on Fridays compared with other weekdays. The study also reported that absenteeism patterns varied according to class period and course type, suggesting that end-of-week absences may differ across instructional contexts and daily school routines.

Evidence from the United Kingdom further suggests that end-of-week absenteeism may be influenced by several behavioral and social factors. A report published by Nesta (2023) indicated that Friday absence rates had already been increasing prior to the pandemic and continued to rise afterward. The report linked these patterns to changing parental attitudes toward the importance of Friday attendance, reduced commitment to regular school attendance at the end of the week, the normalization of elective absences among some families, and the continuation of post-pandemic attendance behaviors and routines.

Other studies have emphasized the social dimension of attendance behavior. Strulik (2010) argued that school attendance may be shaped by community norms and shared social expectations rather than individual factors alone. Kassarnig et al. (2018) similarly found similarities between students’ attendance patterns and those of their social peers. These findings also align with broader ecological perspectives on school absenteeism, which suggest that attendance behavior is shaped not only by individual student factors, but also by institutional routines, school climate, family practices, peer influences, and wider social contexts (Kearney et al., 2022). From this perspective, end-of-week absenteeism may reflect the interaction between temporal school routines and broader behavioral and social influences surrounding school attendance.

Gottfried et al. (2025) further argued that absences occurring on different weekdays may have different educational implications, suggesting that the timing of absences during the school week may matter educationally.

Viewed collectively, these studies suggest that end-of-week absenteeism may be understood through interacting temporal, social, and institutional dimensions. Temporal influences include weekly fatigue, school schedules, holidays, and end-of-week routines. Social influences involve peer expectations, family practices, and the normalization of absenteeism. Institutional influences include school climate, instructional organization, and broader educational routines shaping students’ participation across the school week.

These studies suggest that school attendance may vary systematically across the school week and may be shaped by both temporal organization and broader social context.

### 2.3 School absenteeism in the Saudi context

School absenteeism remains a significant challenge for educational institutions in Saudi Arabia. Although absenteeism is a global educational concern, attendance patterns in Saudi schools appear to reflect distinctive social and temporal characteristics. In recent years, absenteeism has become increasingly concentrated on Thursdays, the final day of the school week. This recurring pattern has been observed across elementary, intermediate, and secondary schools and has attracted growing attention from educators, policymakers, and public discussion.

Public education in Saudi Arabia is compulsory and free across the primary, intermediate, and secondary stages. The school week extends from Sunday to Thursday (National Unified Portal: KSA, 2024). Despite these institutional arrangements, attendance patterns suggest that student disengagement often increases toward the end of the school week. This pattern indicates that attendance behavior may be shaped not only by educational conditions, but also by the temporal organization of schooling and socially shared routines within families and communities.

Research on school absenteeism in Saudi Arabia remains relatively limited. Existing studies nevertheless suggest that absenteeism is influenced by multiple social, educational, and institutional factors. Al-Mujthel (2019) categorized absenteeism factors into school-related influences, such as curriculum design and teacher practices, home-related influences including parental engagement and family routines, and student-related factors such as motivation and behavior. Similarly, AlSayyari and AlBuhairan (2020), in a national study of intermediate and secondary school students, found that absenteeism was associated with social, behavioral, family-related, and health-related factors, highlighting the multidimensional nature of absenteeism within Saudi schools.

Saudi research has also emphasized the importance of school context and institutional conditions in shaping attendance behavior. AlMakadma and Ramisetty-Mikler (2015) found that absenteeism among adolescents in Riyadh was associated with lower levels of school connectedness and parental monitoring. Sideridis and Alamri (2023), drawing on Bronfenbrenner’s ecological framework, similarly reported that student absences were associated with broader school and family characteristics, including school resources, parental education, and school environment. Bennour (2021) also found that school-level contextual conditions were associated with persistent absenteeism and differences in academic outcomes between Saudi schools. These findings suggest that absenteeism in Saudi schools reflects broader institutional and social conditions rather than isolated individual behavior alone.

Public and media discussions have increasingly focused on the concentration of absenteeism on Thursdays. Al-Riyadh (2019) reported statements by the Minister of Education describing Thursday absenteeism as “unacceptable,” reflecting official concern regarding the scale of the phenomenon. Reports published in Okaz (2021) and by Al-Asalani (2019) linked repeated Thursday absenteeism to cumulative weekly fatigue, reduced student motivation, and declining engagement toward the end of the school week, particularly following the introduction of the three-semester academic system. Okaz (2021) also reported that some students used digital communication platforms such as WhatsApp and Telegram to coordinate collective absences before the weekend. Teachers interviewed in these reports further observed that repeated Thursday absenteeism had reduced the effectiveness of the final instructional day in some schools. Additional reports associated absenteeism with limited extracurricular activities, repetitive school routines, and weak parent–school communication (Al-Asalani, 2019; Okaz, 2021). Alharbi (2021) further reported absenteeism rates exceeding 13%, with noticeable increases occurring on particular weekdays, especially Thursdays.

Public discourse also reflects growing concern regarding the normalization of Thursday absenteeism. Al-Madina (2024) reported that some students and families increasingly perceived the final instructional day of the week as less important than other school days. These observations suggest that Thursday absenteeism may function as a socially shared routine shaped by collective expectations surrounding the end of the school week rather than isolated individual decisions.

Despite increasing public and institutional concern, limited research has examined absenteeism in Saudi Arabia from a temporal perspective or explored how weekly routines and institutional time structures shape attendance behavior across the school week. Existing studies have generally examined absenteeism in broad terms without focusing on how attendance patterns vary systematically across particular school days. This gap highlights the need to examine Thursday absenteeism not simply as an individual attendance problem, but as a socially and institutionally patterned phenomenon linked to the organization of school time and end-of-week routines.

### 2.4 Saudi education behavior and attendance rules

The rules of conduct and attendance issued by the Saudi Ministry of Education emphasize the importance of students’ commitment to attending school or approved educational platforms in accordance with the official daily schedule, from the beginning to the end of the school day (Ministry of Education, 2023).

For students from the third grade of primary school through the third grade of intermediate school, attendance is assessed out of 100 points per semester, with the final score representing the average of these evaluations (Ministry of Education, 2023). At the secondary level, students are also assigned 100 marks per academic level, and the cumulative attendance score is displayed on the student’s graduation transcript. Although this score does not contribute to the overall GPA, it is considered a prerequisite for earning first-class honors (Ministry of Education, 2023).

The regulations specify that students who are absent without a valid excuse receive a penalty of half a grade deduction for each missed day during the semester. Additional penalties apply in specific circumstances, including a one-grade deduction for each unexcused absence:

● During the week preceding official vacations,

● Upon returning from vacations, and

● During the week prior to examinations

These regulations indicate that current attendance policies rely primarily on punitive measures to discourage unexcused absences. However, the regulations provide limited emphasis on motivational or engagement-based strategies that encourage regular attendance. Inconsistent implementation of attendance policies across schools may also contribute to recurring absenteeism, particularly during sensitive periods such as the days preceding examinations and on Thursdays.

The updated attendance regulations issued by the Saudi Ministry of Education in 2025 reflect a stronger institutional focus on reducing absenteeism through monitoring systems, attendance grades, and academic penalties. The regulations emphasize procedural accountability through digital attendance tracking and formal excuse submission procedures. They also impose explicit disciplinary measures, such as deducting one attendance grade for each unexcused absence, imposing additional penalties before and after holidays and examinations, and preventing students from academic promotion when unexcused absenteeism exceeds 10% of the academic year (Ministry of Education, 2025).

Despite these efforts, the policies continue to rely primarily on punitive measures, while giving limited attention to motivational or engagement-based approaches. This may reduce their effectiveness in addressing broader social and institutional factors associated with absenteeism, particularly recurring end-of-week absenteeism patterns in Saudi schools. Integrating supportive and incentive-based approaches alongside consistent implementation of attendance policies may strengthen students’ commitment to school attendance and reduce persistent absenteeism.

To provide contextual insight into the structure and distribution of instructional days and holidays during the academic year, the official school calendar is summarized in Table 1.

**TABLE 1:** School calendar for the 2024/2025 academic year in Saudi Arabia.

Date	Event
18/08/2024	Start of the first semester
23/09/2024	National day holiday (2 days)
17/10/2024	Holiday (1 day)
07/11/2024	First semester holiday (10 days)
17/11/2024	Start of the second semester
03/01/2025	Mid-semester holiday (10 days)
20/02/2025	Second semester holiday (12 days)
02/03/2025	Start of the third semester
20/03/2025	Festive holidays (16 days)
06/04/2025	Continuation of studies
04/05/2025	Holiday (1 day)
30/05/2025	Festive holidays (15 days)
15/06/2025	Continuation of studies
26/06/2025	End of the school year vacation

Ministry of Education (n.d), Saudi Arabia. (n.d.) Academic.

The academic calendar for 2024/2025 includes numerous extended holidays distributed across the school year. Frequent interruptions between instructional periods and extended breaks may influence students’ motivation, continuity of learning, and attendance patterns, particularly before and after holidays. Such interruptions may also contribute to reduced engagement toward the end of the school week. These conditions highlight the importance of developing strategies that strengthen student engagement and encourage consistent attendance throughout the academic year.

## 3 Methodology

This study adopted a sequential explanatory mixed-methods design to provide a comprehensive understanding of end-of-week school absenteeism in Saudi schools. In this design, quantitative data were collected and analysed first to identify the major factors associated with Thursday absenteeism, followed by qualitative data to explain and elaborate on the quantitative findings through participants’ perspectives and interpretations (Creswell and Plano Clark, 2018; Tashakkori and Teddlie, 2010).

The quantitative component was used to identify the major factors associated with Thursday absenteeism and to examine general patterns across different respondent groups, including students, family members, and teachers. This phase provided a broad overview of participants’ perceptions regarding the social, institutional, and organizational factors influencing end-of-week absenteeism in Saudi schools.

The qualitative component complemented the quantitative findings by providing deeper insights into participants’ interpretations and explanations of absenteeism at the end of the school week. Through the analysis of open-ended responses, this phase enabled a more detailed understanding of how participants interpreted the temporal, social, and institutional dimensions of Thursday absenteeism.

The integration of the quantitative and qualitative components was achieved at the interpretation stage, where qualitative findings were used to explain and contextualize the statistical patterns identified in the quantitative analysis. This integration enhanced the overall explanatory power of the study and provided a more comprehensive understanding of end-of-week absenteeism as a socially patterned phenomenon.

### 3.1 Data collection

This study examined the phenomenon of Thursday absenteeism in Saudi schools by identifying its underlying causes and contributing factors using a sequential explanatory mixed-methods approach. Both quantitative and qualitative data were collected through an online questionnaire administered via Google Forms.

Participants were recruited using a non-probability convenience sampling approach, which is appropriate when the study targets participants who are readily accessible and directly relevant to the research objectives (Etikan et al., 2016). Consistent with the study design, the research sought to obtain complementary perspectives from the three stakeholder groups most closely associated with school attendance, namely students, parents, and teachers (Creswell and Plano Clark, 2018). Accordingly, 450 questionnaires were distributed equally across the three groups, with 150 questionnaires allocated to each group. A total of 423 completed questionnaires were returned, corresponding to a response rate of 94.0%. Prior to analysis, all returned questionnaires were screened for completeness and data quality. Twelve questionnaires were excluded because they contained substantial missing data or invalid response patterns that rendered them unsuitable for analysis. Consequently, 411 valid questionnaires were retained for the final analysis.

The final analytical sample comprised 77 teachers, 162 parents, and 172 students. Student participants were drawn from intermediate and secondary schools, while teachers represented different school levels. The sample included both male and female participants. To enhance geographical diversity, questionnaires were distributed across the central, eastern, western, northern, and southern regions of Saudi Arabia. Participants were recruited from Riyadh, Makkah, Jeddah, Madinah, Dammam, Qassim, Taif, and Hafr Al-Batin. Participation was voluntary, and the questionnaire was administered online. The study did not distinguish between Saudi nationals and expatriate residents because its primary focus was to examine absenteeism patterns within the broader school context rather than differences related to nationality.

The survey instrument consisted primarily of closed-ended items followed by two open-ended questions. The closed-ended items employed a three-point Likert scale (Agree, Neutral, Disagree) to assess participants’ perceptions of factors influencing Thursday absenteeism. This scale was selected because of its simplicity and its ability to reduce respondent burden while maintaining acceptable reliability (Matell and Jacoby, 1972; Schweizer, 2011; Lucke, 2005).

The questionnaire addressed a range of factors associated with Thursday absenteeism, including institutional scheduling, social coordination, family routines, school environment, motivation, supervision, transportation, and academic workload. Additional items examined participants’ views on potential strategies for improving attendance and restructuring end-of-week educational activities.

The two open-ended questions invited participants to identify additional causes of Thursday absenteeism and suggest possible solutions. These responses provided qualitative insights that were used to explain and contextualize the quantitative findings rather than to develop an independent qualitative inquiry.

The questionnaire was developed by the author following a review of international and Saudi literature on school absenteeism, institutional routines, and student attendance patterns to ensure its contextual relevance. The initial version of the questionnaire was reviewed by academic colleagues with expertise in educational psychology, curriculum and instruction, and educational administration. Their feedback was used to improve the clarity, relevance, and wording of the questionnaire items before pilot testing.

Data collection was conducted between May 2024 and April 2025. Before the main survey, a pilot study involving nine participants (three teachers, three parents, and three students), who were not included in the final sample, was conducted to evaluate the clarity, comprehensibility, and overall usability of the questionnaire. Feedback from the pilot study was used to further refine the wording of several items, particularly those related to the causes of Thursday absenteeism, before the questionnaire was administered in the main study.

The final questionnaire was distributed electronically via email and through direct contact during the researcher’s field visits to schools. All participants were informed about the purpose of the study, the voluntary nature of participation, confidentiality procedures, and their right to withdraw at any time. Electronic informed consent was obtained from all adult participants. For students under the age of 16, parental or legal guardian consent was obtained before participation. The study involved minimal risk and relied exclusively on anonymous responses.

Google Forms was selected as the data collection platform because it facilitated anonymous participation, efficient distribution across multiple regions, and secure password-protected storage of responses. No personally identifying information was collected, and access to the dataset was restricted to the researcher.

### 3.2 Data analysis

Quantitative and qualitative data were analysed separately and integrated during the interpretation stage. Quantitative data were analysed using descriptive statistics, including frequencies, means, standard deviations, and Relative Importance Index (RII) values to identify and rank the factors associated with Thursday absenteeism and the proposed solutions. Comparative analyses across respondent groups were also conducted using the Kruskal–Wallis test.

Qualitative responses obtained from the open-ended questions were analysed using thematic analysis procedures involving coding, categorization, and theme development. The qualitative findings were used primarily to explain and contextualize the quantitative results.

### 3.3 Ethics approval and consent to participate

This study was approved by the Local Research Ethics Committee for Research Involving Living Creatures at the University of Hafr Al Batin, Saudi Arabia. Participation in this study was voluntary, and informed consent was obtained from all participants prior to data collection. For participants under the age of 16, informed consent was obtained from their parents or legal guardians. All procedures performed in this study were in accordance with the ethical standards of the institutional research committee and with the 1964 Declaration of Helsinki and its later amendments. All responses were collected anonymously and used solely for research purposes.

### 3.4 Quantitative analysis

The quantitative data obtained from the three-point Likert scale were analysed using descriptive statistical techniques in SPSS. Frequencies and percentages were calculated to determine the level of agreement among respondents regarding the factors influencing Thursday absenteeism. In addition, means and standard deviations were computed to describe the central tendency and variability of the responses.

To prioritize the factors influencing Thursday absenteeism and the proposed solutions, the study employed the Relative Importance Index (RII). This method is commonly used in social and management research to rank factors according to their perceived importance when analysing ordinal data derived from Likert-scale responses (Khatib et al., 2020; Alaghbari et al., 2019).

The Relative Importance Index (RII) was calculated using the following formula:

RII = ΣW / (A × N)

Where:

W represents the weight assigned by respondents (1 = Disagree, 2 = Neutral, 3 = Agree).

A represents the highest possible weight (*A* = 3).

N represents the total number of respondents.

The resulting RII values range between 0 and 1, where higher values indicate greater perceived importance of a given factor.

#### 3.4.1 Relative importance of factors affecting end-of-week absenteeism

The Relative Importance Index (RII) results presented in Table 2 reveal a patterned configuration of factors shaping absenteeism on the final day of the school week (Thursday). These factors reflect the interaction of institutional time arrangements, collective social coordination, family practices, and school-level organizational conditions.

**TABLE 2:** Questionnaire distribution, response rate, and final analytical sample.

Stage	Description	*n*	%
**Questionnaires distributed**	Students	150	33.3
	Parents	150	33.3
	Teachers	150	33.3
	Total distributed	**450**	**100**
**Returned questionnaires**	Completed questionnaires	423	94.0
**Unreturned questionnaires**	Not returned	27	6.0
**Excluded questionnaires**	Incomplete or invalid responses	12	2.8
**Final analytical sample**	**Students**	**172**	**41.8**
	**Parents**	**162**	**39.4**
	**Teachers**	**77**	**18.7**
	**Total analysed**	**411**	**100**

The response rate was calculated based on the total number of questionnaires distributed (*N* = 450). The percentage of excluded questionnaires was calculated based on the number of returned questionnaires (*N* = 423). Bold values indicate the highest value in each column.

The most influential factor identified was the implementation of the three-semester system, which received the highest relative importance score (89%). Respondents consistently associated this system with cumulative weekly fatigue, intensified academic demands, and disruptions to established weekly rhythms. These findings suggest that the temporal structure of the school year plays a significant role in shaping attendance behavior toward the end of the week.

The second highest-ranked factor was students’ coordination of absences through social media platforms (81%). Participants indicated that communication tools such as WhatsApp and Snapchat facilitate collective decision-making about attendance, reinforcing shared expectations regarding Thursday absenteeism. Two additional factors ranked at similar levels—the influence of absenteeism in neighboring schools (76%) and students’ perception that skipping Thursdays has become a routine practice (76%)—further illustrate how attendance behavior may be shaped by collective social norms rather than isolated individual decisions.

Several mid-ranked factors highlighted the interaction between institutional arrangements and family practices. The absence of sports and recreational activities on Thursdays (74%), weekend travel (74%), and family or social gatherings (71%) were frequently cited as contributing to absenteeism at the end of the school week. Participants also noted that family involvement plays an important role in shaping attendance patterns, with family coordination of absences (71%) and weak encouragement of attendance (68%) reinforcing the normalization of Thursday absenteeism.

Institutional conditions within schools also emerged as relevant influences. Respondents pointed to an unattractive school environment (65%), the presence of multiple breaks within a semester (64%), and weak enforcement of attendance regulations (63%) as factors affecting participation toward the end of the week. These findings suggest that organizational consistency and the quality of the school environment influence students’ willingness to attend school on Thursdays.

Lower-ranked, yet still notable, factors included heavy academic workload (58%), teachers conveying that Thursday attendance is less important (55%), and teacher absenteeism (50%). These results indicate that instructional practices and supervision may influence how students perceive the importance of attending school on the final day of the week.

Additional logistical and personal factors—such as transportation difficulties (52%), family-related problems (51%), medical reasons (54%), and peer bullying (54%)—were also reported. However, these factors exerted comparatively less influence than structural and socially coordinated drivers of absenteeism.

The RII results suggest that absenteeism at the end of the school week reflects a patterned interaction between institutional time arrangements, collective social coordination, organizational practices, and individual circumstances. The prominence of temporally structured and socially coordinated factors indicates that Thursday absenteeism operates as a recurring social pattern shaped by the weekly organization of schooling rather than isolated acts of non-attendance. The prioritization of these factors is summarized in Table 3.

**TABLE 3:** Relative importance of factors associated with end-of-week absenteeism.

Reason	Rank
Implementation of the three-semester system.	89%
Students coordinating absences via social media (WhatsApp, Snapchat, etc.).	81%
Students being influenced by absenteeism in neighboring schools.	76%
Students’ belief that missing Thursday is a persistent and necessary habit.	76%
Lack of sports and recreational activities at school.	74%
Travel and trips during the weekend.	74%
Family and social gatherings occurring on the weekend.	71%
Families coordinating with each other to keep their children absent.	71%
Lack of motivation for students who attend on Thursday.	70%
The high number of short and extended holidays within a semester.	68%
Families’ weak encouragement for their children to attend on Thursday.	68%
The school environment is unprepared and unattractive.	65%
The existence of numerous weekly breaks between semesters.	64%
Weak enforcement of the behavior and attendance policy for Thursday absences.	63%
Weak family-school communication.	59%
Excessive school workload and assignments.	58%
Teachers implying that attendance on Thursday is unimportant.	55%
Medical and health reasons.	54%
Students threatening and bullying their present peers.	54%
Poor performance by teachers in fulfilling their duties on Thursday.	53%
Difficulty with transportation and school buses.	52%
Family reasons and domestic problems.	51%
Absence of teachers from classes on Thursday.	50%

#### 3.4.2 Relative importance of suggested solutions to end-of-week absenteeism

The Relative Importance Index (RII) results presented in Table 3 reveal a clear prioritization of strategies proposed by participants to address absenteeism at the end of the school week. The findings suggest that respondents favor approaches that reorganize the structure, activities, and learning environment associated with Thursdays rather than eliminating the school day altogether.

The highest-ranked solution was improving school infrastructure, which received an importance score of 90%. Participants emphasized the importance of providing facilities such as sports fields, gyms, laboratories, and workshops. These responses indicate that the physical learning environment plays a significant role in shaping students’ willingness to attend school, particularly when the final day of the week competes with alternative leisure opportunities.

A second cluster of highly ranked solutions focused on external partnerships and incentive-based initiatives. Activating partnerships that offer discounts or rewards for regular attendance (86%) and collaborating with external organizations to provide skill-based training opportunities (85%) were viewed as effective strategies. Respondents associated these initiatives with increased motivation and enhanced value attached to end-of-week participation in schooling.

Participants also expressed strong support for restructuring Thursdays around experiential and socially engaging forms of learning. Highly ranked suggestions included dedicating Thursdays to field visits (84%), vocational learning activities (84%), volunteer work and life skills development (83%), and small group projects (83%). These preferences suggest that students and stakeholders perceive experiential and collaborative activities as more appropriate forms of engagement at the end of the school week.

Additional solutions receiving relatively high importance scores included academic competitions (82%), cultural and heritage activities (81%), inter-school competitions (80%), and sports, entertainment, and arts-based activities (79%). Collectively, these findings indicate that participants favor opportunities for interaction, creativity, recognition, and collective participation when reimagining the final day of the school week.

In contrast, the lowest-ranked solutions involved canceling Thursday classes altogether (70%) or converting Thursday into a distance-learning day (68%). These results indicate limited support for eliminating in-person schooling on Thursdays. Instead, participants expressed a preference for maintaining Thursday as a school day while transforming its structure and activities.

Overall, the RII findings suggest that proposed solutions prioritize the reorganization of time, space, and activity on Thursdays rather than the removal of the school day itself. Stakeholders’ preferences emphasize revitalizing end-of-week schooling through environmental improvements, experiential learning, and incentive-based engagement. The prioritization of these solutions is summarized in Table 4.

**TABLE 4:** Relative importance of suggested solutions to end-of-week absenteeism

Proposed solution	Rank
Improving the school’s infrastructure (fields, gyms, labs, workshops) to create an attractive environment.	90%
Activating partnerships with the public/private sectors to offer discounts or prizes for regular attendees.	86%
Activating partnerships to train students on necessary skills.	85%
Dedicating Thursday to visits to museums, factories, companies, and businesses.	84%
Dedicating Thursday to vocational work (blacksmithing, carpentry, IT, cooking, etc.).	84%
Dedicating Thursday to volunteer programs and life skills development.	83%
Dedicating Thursday to small, innovative group projects evaluated at semester’s end.	83%
Dedicating Thursday to academic competitions with encouraging incentives.	82%
Dedicating Thursday to school theater, culture, and national heritage.	81%
Dedicating Thursday to competitions between schools in the region or city.	80%
Dedicating Thursday entirely to sports, entertainment, and artistic activities.	79%
Canceling Thursday classes and adding an extra period to other days.	70%
Converting the fifth school day to distance learning via educational platforms.	68%

### 3.5 Qualitative analysis

The qualitative component of this study employed thematic analysis to examine stakeholders’ perceptions of the factors contributing to Thursday absenteeism in Saudi schools. Open-ended responses were reviewed and coded to identify recurring ideas and patterns within the dataset. These codes were then organized into broader conceptual themes. The qualitative data were analysed through coding, categorization, and theme development procedures consistent with thematic analysis approaches (Braun and Clarke, 2006).

The themes were refined and clearly defined to ensure that they accurately reflected participants’ responses. Illustrative participant statements were used to contextualize and support each theme. The analysis identified eleven major themes describing the key factors associated with fifth-day absenteeism.

NVivo 12 software was used to support the coding and analytical process, enhancing the transparency and analytical consistency of the qualitative analysis. The themes identified through this process are summarized in Table 5.

**TABLE 5:** Themes identified in the data.

Themes	Sentences	*n*	%
School day structure and scheduling	“The length of the school day and the three-semester system are exhausting for students and teachers.” (Student and teacher perspectives) “Because the three-semester system is tiring.” (Student and teacher perspectives) “There are too many interrupted holidays during the school year. Whenever we try to continue the curriculum consistently, another break begins.” (Teacher perspective) “We study throughout the entire year, and the school schedule feels exhausting.” (Student perspective)	68	16.56%
Physical and mental fatigue	“By Thursday, students feel physically and mentally exhausted from the school week.” (Teacher perspective) “I feel tired and lose motivation to attend school by the end of the week.” (Student perspective) “Students become exhausted because of the long school routines and the educational environment.” (Parent perspective)	49	11.91%
Socially coordinated and pre-arranged absenteeism	“Many students agree in advance to be absent on Thursdays.” (Teacher perspectives) “Some parents are aware of these absence arrangements and do not object to them.” (Teacher perspective) “Students often discuss Thursday absences with each other before the weekend.” (Student perspective) “Some families communicate with each other regarding Thursday absences.” (Parent perspective) “We have a WhatsApp group where we regularly discuss who will attend school on Thursday, and if attendance is expected to be low, many students decide not to go.” (Student perspective)	87	21.16%
Educational environment	“The school environment does not encourage students to attend regularly.” (Parent perspective) “Overcrowded classrooms and the weak educational environment reduce students’ motivation to attend school.” (Teacher perspective) “The long school day and the poor educational environment make students less willing to attend on Thursdays.” (Parent perspective)	37	9.00%
Transportation and logistics	“Some students are absent because of travel plans and transportation difficulties.” (Parent perspective) “The long distance between home and school affects students’ willingness to attend on Thursdays.” (Teacher perspective) “The long school day and transportation problems make attending school more difficult at the end of the week.” (Parent perspective) “We sometimes travel before the weekend, which makes Thursday attendance difficult for my son.” (Parent perspective)	23	5.59%
Academic workload	“The long school day and the heavy workload place pressure on students by the end of the week.” (Parent perspective) “The number of school subjects and assignments becomes exhausting for students toward Thursday.” (Student perspective) “By Thursday, I feel overwhelmed by the number of subjects, assignments, and exams.” (Student perspective) “Some assignments are given in a punitive way, which makes students less motivated to attend.” (Parent perspective)	36	8.76%
Supervision and follow-up	“Most parents are at work, so it is difficult to monitor students’ attendance regularly.” (Parent perspective) “The long school day and weak follow-up reduce students’ commitment to attendance.” (Teacher perspective) “Some students are absent because there is limited parental follow-up regarding school attendance.” (Teacher perspective) “There is insufficient monitoring of absenteeism at different supervisory levels.” (Teacher perspective)	26	6.32%
Motivation and engagement	“Some students are no longer interested in attending school regularly.” (Teacher perspective) “I sometimes feel unmotivated to attend school, especially at the end of the week.” (Student perspective) “By the end of the week, I feel mentally exhausted and less motivated to attend school.” (Student perspective) “There is limited encouragement from school administration to motivate students to attend on Thursdays.” (Parent perspective)	24	5.84%
Awareness and cultural factors	“Some families do not view Thursday attendance as important.” (Teacher perspective) “There is limited community awareness about the importance of regular school attendance.” (Teacher perspective) “Some students do not fully recognize the importance of attending school consistently.” (Teacher perspective)	19	4.62%
Teacher-related factors	“Frequent teacher absences affect students’ commitment to regular attendance.” (Parent perspective) “Some teachers lack effective teaching and classroom engagement skills.” (Parent perspective) “The limited presence of some teachers at school reduces students’ motivation to attend.” (Student perspective) “Half of the classes are empty on Thursdays, and many teachers are absent.” (Student perspective) “We usually do not study new lessons on Thursdays.” (Student perspective)	27	6.58%
Institutional policies	“Some school procedures waste students’ time and reduce the importance of attending on Thursdays.” (Parent perspective) “Some assignments are given in a punitive way rather than for learning purposes.” (Student perspective) “There is limited encouragement from school administration to motivate students to attend regularly.” (Parent perspective)	15	3.65%

#### 3.5.1 School day structure and scheduling

School day structure and scheduling emerged as a major contributor to Thursday absenteeism, accounting for 16.56% of participants’ responses. Respondents consistently referred to the length of the school day, the limited weekend, and particularly the three-semester system as overly demanding for both students and teachers. Extended instructional hours and compressed schedules were described as generating cumulative fatigue, which was perceived to reduce students’ willingness to attend school, especially toward the end of the week.

These accounts indicate that absenteeism is closely linked to the temporal organization of schooling, where daily schedules and weekly structures combine to influence participation. Similar patterns have been documented in international research linking extended instructional hours, fatigue, and reduced student engagement with increased absenteeism (Askeland et al., 2015; Klein and Sosu, 2022).

#### 3.5.2 Physical and mental fatigue

Physical and mental fatigue accounted for 11.91% of responses, with participants consistently emphasizing the cumulative exhaustion experienced by students, teachers, and even parents by the end of the week. Statements such as “fatigue at the end of the week” and “exhaustion from the school environment” illustrate this growing burden. Respondents noted that the length of the school day intensifies fatigue, diminishing students’ motivation to attend on Thursdays. Several student participants also described feeling mentally exhausted and less motivated to attend school by the end of the week. These findings align with studies linking long school hours and accumulated tiredness to lower attendance and reduced academic engagement (Smerillo et al., 2018; Klein and Sosu, 2022).

#### 3.5.3 Socially coordinated and pre-arranged absenteeism

The most prominent theme identified in the qualitative findings was socially coordinated and pre-arranged absenteeism, accounting for 21.16% of participants’ responses. Respondents frequently described a coordinated pattern in which students—and in some cases parents—agree in advance not to attend school on Thursdays. This form of absenteeism was commonly portrayed as planned rather than spontaneous, with decisions often made prior to the school day.

Participants reported that digital communication platforms, particularly WhatsApp and Telegram groups, played a central role in facilitating and normalizing these coordinated absences. Through these platforms, students shared information, reinforced shared expectations, and collectively confirmed non-attendance, contributing to a sense that Thursday absenteeism was both anticipated and socially endorsed. In several accounts, parental awareness or acceptance further reinforced these practices.

Student accounts further illustrated how absenteeism was collectively negotiated through peer communication. Some students described using WhatsApp groups to discuss expected attendance levels on Thursdays and deciding collectively whether to attend school.

These findings indicate that absenteeism at the end of the school week operates as a socially organized pattern rather than an individual act of non-attendance. Attendance behavior on Thursdays was described as shaped by peer influence, shared expectations, and collective coordination embedded within social networks. Similar patterns have been noted in previous research, which highlights the influence of social dynamics and community norms in shaping attendance behavior (Rocque et al., 2017; Daraganova et al., 2014). Recent research has also emphasized the growing role of digital communication and social media platforms in shaping adolescents’ social interactions, behavioral coordination, and collective practices (Falcón-Linares et al., 2023).

#### 3.5.4 Educational environment

Representing 9% of responses, the educational environment was identified as another influential factor. Participants criticized the lack of stimulating learning environments, overcrowded classrooms, and limited engagement strategies—particularly toward the end of the week when fatigue is higher. Statements such as “the educational environment is not stimulating” and “overcrowding reduces motivation” highlight how the physical and instructional environment plays a role in absenteeism. These findings are consistent with research showing that unengaging school environments contribute to reduced attendance and diminished academic interest (Klein and Sosu, 2022; Askeland et al., 2015).

#### 3.5.5 Transportation and logistics

Transportation and logistical difficulties, accounting for 5.59%, include long distances between home and school and limited transportation options. Respondents frequently referenced the burden of commuting, particularly when combined with accumulated fatigue, making Thursday attendance more difficult. These challenges intensify toward the end of the week, serving as a practical barrier to consistent attendance.

#### 3.5.6 Academic workload

Academic workload contributed 8.76% of responses. Many participants cited heavy assignments, frequent exams, and repetitive subjects as reasons students avoid attending on Thursdays. Some described assignments as punitive in nature, creating additional stress. These insights echo research showing that students may skip school to avoid academic pressures or assessments scheduled late in the week (Ansari et al., 2020; Klein and Sosu, 2022; Cattan et al., 2023).

#### 3.5.7 Supervision and follow-up

Weak supervision and follow-up accounted for 6.32% of responses. Participants emphasized insufficient parental monitoring, along with limited oversight from teachers and school administrators. Comments such as “parents are at work” and “lack of follow-up” suggest that reduced accountability enables absenteeism—particularly when absences are pre-arranged.

#### 3.5.8 Motivation and engagement

This theme, representing 5.84%, highlights the central role of student motivation in attendance. Respondents reported a “lack of interest in learning,” “low motivation,” and limited administrative encouragement. End-of-week fatigue further diminishes engagement. These findings align with studies emphasizing the importance of both intrinsic and extrinsic motivation in sustaining regular attendance (Liu et al., 2021).

#### 3.5.9 Awareness and cultural factors

Awareness and cultural factors (4.62%) reflect broader societal attitudes toward attendance. Respondents pointed to insufficient community awareness, weak educational culture, and parental perceptions that minimize the importance of regular attendance—particularly on Thursdays. These findings illustrate how cultural norms and family beliefs shape attendance behaviors.

#### 3.5.10 Teacher-related factors

Teacher-related issues accounted for 6.58% of responses. These included teacher absences, limited instructional skills, and strained student–teacher relationships. Respondents highlighted concerns such as “teacher absence at school” and “student fear of teachers.” Some student participants also noted that Thursday classes were often partially empty and that new instructional material was rarely introduced at the end of the week. Such factors negatively influence students’ willingness to attend, consistent with findings by Ansari et al. (2020) and Klein and Sosu (2022).

#### 3.5.11 Institutional policies

The least frequent theme (3.65%) concerned institutional policies. Respondents criticized punitive approaches, inflexible attendance requirements, and insufficient administrative encouragement. Some referenced “punishment-based assignments” and a lack of supportive policies that address underlying causes of absenteeism. These findings resonate with research suggesting that rigid, punitive policies can inadvertently worsen attendance issues (Ozcan, 2022).

### 3.6 Comparative analysis across respondent groups

To examine potential differences across respondent groups, comparative analyses were conducted between students, family members, and teachers regarding overall perceptions of the factors associated with fifth-day absenteeism. Mean scores and standard deviations were calculated based on participants’ overall responses to the questionnaire items. A Kruskal–Wallis test was then conducted to examine whether statistically significant differences existed across respondent groups.

A Kruskal–Wallis test revealed no statistically significant differences between respondent groups (*H* = 1.14, *p* = 0.566), suggesting that students, family members, and teachers shared relatively similar overall perceptions regarding the factors associated with fifth-day absenteeism. However, Table 6 presents descriptive comparisons intended to illustrate how specific absenteeism-related factors were perceived differently across stakeholder groups.

**TABLE 6:** Comparative analysis across respondent groups.

Respondent group	*N*	Mean	SD
Students	172	2.18	0.29
Family members	162	2.13	0.30
Teachers	77	2.13	0.34

The comparative analysis revealed several interpretive differences across respondent groups (Table 7). Teachers expressed stronger concern regarding weak family encouragement, socially coordinated absenteeism through digital communication platforms, weekend travel, and the repeated disruption associated with extended holidays. Family members also reported relatively high perceptions regarding reduced student motivation, the structure of the academic calendar, and the influence of recreational and social routines at the end of the week. In contrast, students more frequently associated absenteeism with transportation difficulties and teacher-related classroom practices. Family members generally occupied an intermediate position between students and teachers across most factors. These findings suggest that stakeholders interpret Thursday absenteeism through different institutional and social perspectives, even when overall perceptions remain broadly consistent across groups.

**TABLE 7:** Selected differences across stakeholder groups regarding causes of Thursday absenteeism.

Factor	Students mean	Family members mean	Teachers mean
Families’ weak encouragement for their children to attend on Thursday.	1.85	1.96	2.39
Students coordinating absences via social media (WhatsApp, Snapchat, etc.).	2.39	2.25	2.52
Lack of motivation for students who attend on Thursday.	2.25	2.27	1.92
The high number of short and extended holidays within a semester.	1.89	2.06	2.22
Travel and trips during the weekend.	2.14	2.09	2.42
Difficulty with transportation and school buses.	1.71	1.62	1.39
Poor performance by teachers in fulfilling their duties on Thursday.	1.74	1.75	1.43
Absence of teachers from classes on Thursday.	1.65	1.62	1.32

## 4 Discussion

The findings of this study suggest that absenteeism at the end of the school week in Saudi Arabia is not a random or isolated phenomenon. Rather, it represents a patterned social practice shaped by the temporal organization of schooling, institutional routines, and shared social expectations. This interpretation is consistent with recent scholarship that views problematic school absenteeism as the outcome of interacting institutional, social, psychological, and organizational influences (Harling and Strandler, 2025). Previous research has similarly described absenteeism as a multifaceted issue shaped by contextual and educational conditions (Rocque et al., 2017; Ressa and Andrews, 2022; Tonge and Silverman, 2019). The present study extends this literature by showing how these factors converge around a specific temporal boundary: Thursday, the final day of the Saudi school week. This interpretation is also consistent with findings from Saudi schools indicating that absenteeism and academic outcomes are shaped by interconnected contextual and institutional conditions (Sideridis and Alamri, 2023). The findings further align with broader evidence suggesting that school absenteeism reflects cumulative social and educational processes rather than isolated individual behavior (Askeland et al., 2015).

A central contribution of this study is its emphasis on social coordination. The prominence of pre-arranged absenteeism suggests that Thursday absenteeism is not merely a matter of individual non-compliance. Instead, it operates through collective expectations and shared decision-making. Digital communication platforms, including WhatsApp and Telegram, appear to function as social infrastructures through which absence is discussed, coordinated, and normalized. In this context, absenteeism becomes embedded in everyday peer and family interactions, reflecting broader intersections between communication, time, and social life. This finding is consistent with research highlighting the influence of peer networks and social norms on attendance behavior (Rocque et al., 2017; Daraganova et al., 2014). It also suggests that digital connectivity may intensify the coordination of end-of-week withdrawal from school obligations. The collective nature of Thursday absenteeism is further supported by evidence indicating that absenteeism may operate as a shared classroom phenomenon rather than solely an individual educational risk. Gottfried and Ansari (2022) found that higher levels of classroom absenteeism were associated with lower academic achievement and weaker executive functioning among students.

The findings also highlight the importance of institutional time in shaping school participation. Participants frequently linked Thursday absenteeism to the structure of the school day, cumulative weekly fatigue, and the organization of the academic calendar. Fatigue, therefore, should not be understood only as an individual condition. It also reflects the accumulation of institutional demands across the school week. Thursday appears to function as a temporal threshold, marking a gradual disengagement from school routines before the weekend.

Similar end-of-week attendance patterns have been reported internationally. Hollon et al. (2025) linked higher Monday and Friday absence rates to cumulative weekly fatigue, weekend-related family routines, and elective absences before holidays. The study also highlighted changing attendance behaviors following the COVID-19 pandemic.

Clifton-Sprigg and James (2025) similarly found that Friday absences in England were associated with families extending holidays before school breaks and attempts to avoid travel congestion during peak holiday periods. Their findings further suggested that end-of-week absenteeism was more common in areas characterized by persistent absenteeism and socioeconomic deprivation.

Nesta (2023) additionally reported that changing parental attitudes toward Friday attendance and the normalization of elective absences contributed to increasing end-of-week absenteeism patterns in the United Kingdom.

These findings suggest that attendance behavior may be shaped by broader temporal, social, and family routines rather than by isolated individual decisions.

This interpretation also supports international research demonstrating that extended instructional hours and rigid schedules may undermine motivation and contribute to end-of-week disengagement (Smerillo et al., 2018; Klein and Sosu, 2022). The significance of end-of-week absenteeism extends beyond the immediate loss of instructional time. Previous research has shown that recurring absenteeism is associated with lower academic achievement, weaker educational engagement, and reduced social participation (Gottfried, 2014). Consequently, the normalization of Thursday absenteeism may have implications that extend beyond attendance itself, potentially influencing students’ long-term educational experiences and levels of school engagement.

The normalization of Thursday absenteeism also reflects the interaction between schooling and social life beyond the school environment. Family routines, weekend travel, and social gatherings were often prioritized over Thursday attendance. This indicates that school attendance at the end of the week is negotiated within wider family and social expectations. In this sense, absenteeism represents a tension between institutional time and social time rather than a simple rejection of schooling.

This interpretation is consistent with findings by Clifton-Sprigg and James (2025), who associated end-of-week absenteeism with holiday extension and family travel before school breaks. Hollon et al. (2025) similarly linked higher end-of-week absences to weekend-related family routines and elective absences before holidays. Nesta (2023) further reported changing parental attitudes toward Friday attendance and the growing normalization of elective absences among some families.

Teachers and family members tended to frame Thursday absenteeism as a problem of educational responsibility and institutional commitment, whereas students more often associated it with fatigue, routine, and the approaching weekend.

The quantitative findings reinforce this interpretation. The three-semester system emerged as the most influential factor associated with Thursday absenteeism, suggesting that fragmented academic schedules and intensified instructional demands may disrupt established temporal rhythms within schools. Similarly, the high ranking of socially coordinated absenteeism and the perception of Thursday as a habitual non-school day indicate that attendance behavior is shaped by collective expectations.

Nesta (2023) suggested that end-of-week absenteeism may become normalized through repeated family routines and changing attitudes toward school attendance. Hollon et al. (2025) also linked end-of-week absences to elective absence practices surrounding weekends and holidays.

These findings are consistent with international evidence showing that absenteeism varies systematically across the school week, particularly toward the final instructional day (Clifton-Sprigg and James, 2025; Kirksey et al., 2023). They also resonate with studies emphasizing the role of institutional time regimes in structuring participation and withdrawal across organizational settings (Alharbi, 2021).

The findings further suggest that punitive or purely administrative responses are unlikely to address the problem effectively. Such responses may overlook the temporal and social meanings attached to Thursday absenteeism. Participants’ preference for experiential, activity-based, and socially engaging Thursday programmes indicates a desire to reconfigure the meaning of the final school day. Rather than removing Thursday from the school week, stakeholders appear to favor transforming it into a distinct educational space that is more aligned with students’ rhythms, interests, and expectations. The findings additionally suggest that institutional and environmental conditions within schools may contribute to absenteeism patterns. Previous research has demonstrated that positive school climate and students’ perceptions of the educational environment are associated with lower absenteeism (Karlberg et al., 2022).

Beyond explaining the causes of Thursday absenteeism, the present findings highlight the practical value of examining absenteeism from a temporal perspective. Previous research has generally treated absenteeism as a broad educational problem, whereas the present study demonstrates that attendance behavior is also influenced by the organization of the school week and the social meanings attached to particular school days. By integrating the perspectives of students, parents, and teachers, the study provides a broader understanding of how institutional routines, family practices, and shared expectations interact to shape Thursday absenteeism.

These findings have important implications for educational practice. Improving attendance at the end of the school week is unlikely to depend solely on stricter attendance regulations. It also requires school practices that increase students’ engagement and strengthen the educational value of the final instructional day. Participants consistently favored practical and interactive learning experiences over routine classroom instruction. Educational visits, project-based learning, extracurricular activities, community partnerships, and life-skills programmes may therefore encourage regular attendance and strengthen students’ connection with school.

The findings also highlight the importance of strengthening collaboration between schools and families. Parents play an important role in shaping attendance expectations and end-of-week routines. Regular communication and shared responsibility between schools and families may help reduce the social acceptance of Thursday absenteeism while reinforcing the importance of consistent school attendance.

Accordingly, efforts to reduce Thursday absenteeism should combine existing attendance regulations with educational practices that make the final school day more engaging, meaningful, and responsive to students’ learning needs. Such an approach has the potential not only to improve attendance but also to strengthen students’ educational engagement and foster a more positive partnership between schools and families.

From a broader perspective, this study contributes to educational and social research by showing how institutional schedules shape collective behavior. Thursday absenteeism in Saudi schools illustrates how temporal arrangements can produce patterned forms of participation and withdrawal that extend beyond individual choice. Understanding absenteeism through a temporal–social lens highlights the need to examine how weekly rhythms, academic calendars, family routines, and shared social expectations interact to shape everyday practices within schooling systems.

## 5 Limitations

This study was conducted within the Saudi educational context and reflects specific institutional schedules, cultural routines, and social understandings associated with the school week. As a result, the direct transferability of the findings to other educational systems may be limited. The study also relied primarily on participants’ self-reported perceptions and experiences rather than direct behavioral observations or longitudinal attendance records. Consequently, the findings reflect how participants interpret and experience Thursday absenteeism within their social and institutional environments.

In addition, the focus on Thursday as a temporal boundary highlights end-of-week dynamics specific to the organization of schooling in Saudi Arabia. Future research could examine whether similar temporally structured patterns of absenteeism emerge across different institutional, cultural, and educational contexts.

The study did not collect detailed demographic information to permit comparative analyses based on gender or nationality status (Saudi nationals and expatriate residents). Furthermore, the study focused on participants from intermediate and secondary schools and did not include primary school students. Future research could examine how absenteeism patterns vary across demographic groups, educational stages, and student populations within different school contexts.

## 6 Conclusion

This study demonstrates that absenteeism at the end of the school week in Saudi Arabia represents more than individual disengagement or occasional non-attendance. Rather, Thursday absenteeism reflects a socially patterned form of behavior shaped by the temporal organization of schooling and the shared meanings attached to the final day of the institutional week. When students, families, and schools begin to interpret Thursday as a distinct temporal space within the school calendar, attendance patterns gradually shift in response to these shared expectations.

The findings indicate that absenteeism develops progressively across the school week, influenced by cumulative fatigue, established routines, and collective social expectations. By the end of the week, many students appear to experience Thursday not simply as another instructional day, but as the closing phase of an intensive weekly cycle. At the same time, social coordination facilitated through peer networks, family practices, and digital communication reinforces the perception that attendance on the final day of the week is negotiable.

Viewed from this perspective, Thursday absenteeism reflects an interaction between institutional time and everyday social life. The structure of the school week itself shapes how participation is experienced, influencing when attendance is perceived as meaningful and when withdrawal becomes socially acceptable. Understanding absenteeism through this temporal–social lens shifts the discussion beyond individual responsibility and highlights the importance of institutional rhythms in shaping participation within educational systems.

By conceptualizing absenteeism as a temporally structured social practice, this study contributes to broader interdisciplinary discussions concerning how institutional time arrangements influence collective behavior. The findings invite further research into how weekly rhythms, institutional calendars, and shared expectations shape participation patterns across different educational contexts. Attending to how time is experienced within schooling, rather than solely how it is scheduled, may offer new perspectives for addressing persistent challenges related to student engagement and participation.

The findings also suggest that improving attendance on Thursdays may require schools to rethink how the final day of the week is organized and experienced by students. Creating more engaging and socially meaningful school environments through activity-based learning, sports programmes, practical workshops, collaborative projects, and extracurricular activities may strengthen students’ sense of participation and connection to school. Making Thursday a more active and attractive educational experience may help reduce the normalization of absenteeism at the end of the school week.

## Data Availability

The raw data supporting the conclusions of this article will be made available by the authors, without undue reservation.
